# Cerebral Small Vessel Disease: Neuroimaging Features, Biochemical Markers, Influencing Factors, Pathological Mechanism and Treatment

**DOI:** 10.3389/fneur.2022.843953

**Published:** 2022-06-14

**Authors:** Beida Ren, Ling Tan, Yuebo Song, Danxi Li, Bingjie Xue, Xinxing Lai, Ying Gao

**Affiliations:** ^1^Department of Neurology, Dongzhimen Hospital, Beijing University of Chinese Medicine, Beijing, China; ^2^Institute for Brain Disorders, Beijing University of Chinese Medicine, Beijing, China; ^3^Chinese Medicine Key Research Room of Brain Disorders Syndrome and Treatment of the National Administration of Traditonal Chinese Medicine, Beijing, China; ^4^Department of Cardiology, Xiyuan Hospital, China Academy of Chinese Medical Sciences, Beijing, China

**Keywords:** cerebral small vessel disease, neuroimaging, biological markers, pathological mechanism, treatment, research difficulties, future development direction

## Abstract

Cerebral small vessel disease (CSVD) is the most common chronic vascular disease involving the whole brain. Great progress has been made in clinical imaging, pathological mechanism, and treatment of CSVD, but many problems remain. Clarifying the current research dilemmas and future development direction of CSVD can provide new ideas for both basic and clinical research. In this review, the risk factors, biological markers, pathological mechanisms, and the treatment of CSVD will be systematically illustrated to provide the current research status of CSVD. The future development direction of CSVD will be elucidated by summarizing the research difficulties.

## Introduction

CSVD refers to a group of clinical, cognitive, imaging, and pathological manifestations caused by structural and functional changes in small vessels including small arteries, arterioles, small venules, venules, and capillary syndrome (1). CSVD is a systemic and whole-brain chronic disease with high recurrence and low mortality rates. Histopathological studies have shown that a narrowed lumen and stiffer walls of the affected vessel hinder blood perfusion and transmural gas transfer (2). There are four main clinical manifestations of CSVD: stroke, cognitive impairment, recurrent lacunar infarction, and white matter lesions (WMLs). Specifically, acute-phase lesions are prone to lacunar infarction and cerebral parenchymal hemorrhage, accounting for 20% of all symptomatic stroke types (3, 4), and chronic-phase lesions show low perfusion and cause persistent and progressive damage to the corresponding brain tissue, including cognitive impairment and dementia, abnormal gait, dyskinesia, urinary retention, and emotional and personality disorders. Additionally, CSVD causes significant vascular depression. It predisposes individuals to emotional, behavioral, and personality disorders manifesting as apathy, agitation, lack of motivation, and other conditions (5). The pathological classification of CSVD includes arteriolar sclerosis, cerebral amyloid vascular disease, hereditary cerebral small-vessel disease, inflammation and immune-mediated small-vessel disease, venous collagen disease, and other small-vessel diseases (2).

Epidemiological surveys have revealed that at least 700 million people worldwide experience different forms of CSVD, accounting for 25% of stroke cases, 45% of dementia cases, and 70% of vascular dementia (VD) cases (6). CSVD is also the main cause of neurological loss and cognitive decline in the elderly population. As an age-related risk factor, CSVD contributes to an increase in incidence, accounting for 80% of the total incidence. CSVD is particularly common among individuals over 60 years of age, and its incidence is close to 100% in individuals aged above 90 years (7). With an increasing rate of aging of the population and the high incidence of risk factors for cerebrovascular diseases such as hypertension and hyperlipidemia, the incidence of CSVD and related cognitive dysfunction diseases will increase further. CSVD has drawn the attention of neurologists. According to statistics from China, CSVD accounts for 46% of all ischemic stroke causes, of which lacunar infarction accounts for 25–50% of cases (8); for 11–25% of cerebral hemorrhage cases among healthy elderly individuals without cognitive impairment; for 87% of WMLs among people aged 60–70 years; and for almost 100% among people aged 80–90 years (9). Additionally, sex differences are closely related to cerebrovascular disease. In China, the detection rate of CSVD among males is 71.42%, which is significantly higher than that of females (28.58%) (10).

We present a review of seven aspects: neuroimaging characteristics, biomarker classification, influencing factors, pathological mechanisms, treatment status, research difficulties, and research prospects. The review also details the neuroimaging characteristics of CSVD, systematically summarizes the classification and characteristics of CSVD biomarkers, further discusses the challenges in current research on CSVD, and provides a clear understanding of the research direction of CSVD basis.

## Neuroimaging Of Csvd

### Classification of Neuroimaging

The standards for reporting vascular changes in neuroimaging studies published in 2013 have affected the diagnostic criteria. CSVD mainly includes recent small subcortical infarctions, presumed vascular-derived lacunar infarction (LI), presumed vascular-derived white matter hyperintensities (WMHs), enlarged perivascular space (EPVS), cerebral microbleed (CMB), and brain atrophy (8). The detection rates of WMHs, LI, and CMBs among the elderly are 50–98%, 8–28, and 5%, respectively (11, 12). The overall data of China are higher than those of European and American countries (13).

### Features of Neuroimaging

Traditional imaging examinations have a limited effect: ① computed tomography (CT) and CT angiography (CTA) are helpful for the differential diagnosis of basal ganglia or cerebral vascular wall calcification. However, CT findings indicate that, limited by the onset time and lesion size, the appearance and severity of clinical symptoms and signs are mainly determined by the lesion site and size. ② As the gold standard for diagnosing vascular diseases related to the central nervous system, digital subtraction angiography (DSA) can display smaller arteries, including perforating arteries but fails to clearly display blood vessels with a diameter of < 850 μm. DSA is an invasive examination accompanied by ionizing radiation, which is the reason for its limited clinical diagnostic value (14) (Table 1).

**Table 1:** Classification of imaging diagnosis of CSVD.

**Development** **stage**	**Imaging method**	**Feature**
Traditional imaging	CT/CTA	(1) Differential diagnosis of basal ganglia and cerebral vascular wall calcification; (2) Limited diagnostic value for CSVD.
	DSA	(1) Showing small arteries including perforating arteries; (2) Blood vessels with a diameter less than 850μm cannot be clearly displayed; (3) Invasive examination; (4) Lonizing radiation.
Currently using	MRI-T1/T2/FLAIR/DWI/GRE/SWI	CSVD is considered when the total score is≥2: (1) Number of vascular-derived lacunas≥1; (2) The number of CMB in the deep or under the tentorium≥1; (3) EPVS (Level 2-4); WMH around the ventricle: Fazekas scale score≥3 points or deep≥2 points.
Further development	UHF	(1) Visualization; (2) No contrast agent; (3) measuring the blood flow velocity through the artery; (4) Poorly visible information about microvessels can be obtained.
	rs-fMRI	(1) Judging the network connection between various functionally related brain areas, which is helpful for the early differential diagnosis of CSVD.
	TA	(1) Suitable for white matter and brain volume; (2) Predicting onset of dementia; (3) Potential ofmarker
	DTI/PSMD/DSEG	(1) DTI: White matter microstructure changes; (2) PSMD: A hopeful surrogate indicator for observing the progress of CSVD; (3) DSEG-θ: An accurate method to assess brain microstructure damage; identifying the risk of dementia inpatients with CSVD; indicating clinical severity; Preclinical markers.
	ASL	(1) ASL is highly consistent with the contrast-enhanced MRI display; (2) Low sensitivity to motion artifacts.
	PET	(1) Distinguish between vascular and degenerative cognitive impairment.

Currently, magnetic resonance imaging (MRI) is the most reliable imaging diagnostic method, with epoch-making significance and a guiding role in the early detection, diagnosis, and treatment of CSVD. Commonly used imaging methods include T1, T2, FLAIR, diffusion-weighted imaging (DWI), gradient echo imaging (GRE), and MRI-sensitive weighted imaging (SWI). MRI can detect smaller lacvities, especially by DWI in MRI. Lacunar (old lacunar cerebral infarction): all MRI sequences showed cerebrospinal fluid (CSF)–like signals, that is, low signal on T1 and FLAIR images and high signal on T2 images; new subcortical small infarction (new LI): DWI showed 3–20 mm high-signal lesions. MRI is the gold standard for the diagnosis of Leukoaraiosis (LA). The lesions showed low signal intensity on T1-weighted imaging (WI) and high-signal intensity on T2 WI and FLAIR, which is more sensitive than CT. Diffusion tensor imaging (DTI) can reveal the ultrafine structural changes in white matter fiber bundles, which is helpful for understanding WML sites and cortical functional activities. CMBs are round or quasi-round uniform low signals formed due to signal loss on GRE-T2 ^*^ WI or SWI sequence, with a diameter of 2–10 mm. They neither have an edema zone around them nor a brain parenchymal hematoma. There are MRI total scoring items of CSVD. CSVD will be considered if the total score is more than 2 according to the following items (each worth 1 point, and their sum is the total score): (1) number of vascular-derived lacunar foci ≥1; (2) number of deep or inferior CMBs ≥1; (3) EPVS (grades 2–4); WMH periventricular: Fazekas scale score ≥3 points or deep ≥2 points.

Further development of imaging technology has provided new perspectives and methods for the diagnosis of CSVD. First, ultra-high-field strength (UHF) MRI has achieved remarkable results in the visualization of the CSVD brain microstructure and microvascular imaging (15). Compared with traditional invasive angiography methods, UHF MRI can clarify the diagnosis of peripheral vascular disease and CSVD without the need for contrast agents. Additionally, latest research has proved that 7.0 T MRI can be used to measure the blood flow velocity of the penetrating artery. Moreover, the length, radius, curvature, and other information about the microvessels can be obtained with poor visibility (16). Second, resting state functional MRI, which assesses the network connection between different functionally related brain areas and reflects the brain functional activities in the basic state, is helpful for the early diagnosis of CSVD and has huge application potential (17, 18). Third, texture analysis is based on conventional T1 and FLAIR MRI detection. It is an emerging method for evaluating and diagnosing CSVD and is more suitable for white matter tissue. The biggest advantage lies in the convenience of clinical data collection. Furthermore, it can generate various texture parameters (TP) that contribute to distinguishing between patients with CSVD and control subjects. TP is highly correlated with other MRI parameters such as brain volume and WML volume. After controlling for age, sex, and other parameters, TP was related to executive function and overall function at baseline and can predict the onset of dementia. Studies have shown that TP has the potential to be a marker of CSVD (19). Fourth, DTI, peak width of skeletonized mean diffusivity (PSMD), and fully automatic DTI segmentation technology (DSEG-θ) were performed. DTI is an MRI sequence that can be used to observe microstructural changes in white matter. Measurement methods mainly include fractional anisotropy and mean diffusivity. Some researchers have used the white matter skeleton of the brain to measure the peak width of the average diffusion rate in the brain through DTI parameters to evaluate CSVD, which is called PSMD (20). It eliminates the possibility of errors resulting from CSF contamination and simultaneously improves the sensitivity of CSVD-related changes. Thus, PSMD may be a surrogate indicator for detecting the progression of CSVD. DSEG-θ, based on DTI, also provides an accurate method for evaluating brain microstructural damage in patients with CSVD. It is an important tool for identifying the risk of dementia in patients with CSVD and for detecting the clinical severity of CSVD to a certain extent. The significant advantage of this method is that it provides a more reliable and faster alternative evaluation method without processing. However, a one-to-one comparison of scan results between patients and healthy controls is a notable limitation (21). Fifth, blood perfusion MRI reflects the microvascular distribution, blood perfusion, and tissue hemodynamics. Commonly used sequences include dynamic magnetic-sensitive contrast-enhanced perfusion imaging and arterial spin labeling (ASL) MRI (22). Of these, ASL technology is widely used, including continuous labeling, pulse labeling, and pseudo-continuous arterial self-selection labeling, as recommended in current clinical studies. ASL under UHF strength and contrast-enhanced MRI shows great consistency and a reliable application value. However, sensitivity to motion artifacts is a disadvantage (23). Sixth, compared with positron emission computed tomography (PET), CT and MRI can detect the morphological lesions of CSVD, but they barely determine the underlying pathological changes. Molecular imaging performed using PET has the advantage of distinguishing between vascular and degenerative cognitive impairment. It can detect and quantitatively assess these pathological changes, reveal the individual physiological and pathological mechanisms of patients, and contribute to the proposal of new diagnoses for better treatment strategies (24).

With the rapid development of imaging technology, inspiring progress has been made in the diagnosis of CSVD. However, caution should be exercised when relying entirely on advanced technology to solve relevant clinical problems. Instead, the appropriate sequence or multimodal research, clear judgment criteria, and standardized descriptions should be used in combination to assist researchers in conducting in-depth research on CSVD to answer questions oriented from clinical practice.

## Biochemical Markers and Influencing Factors

Clarifying the relevant factors affecting the onset of CSVD can contribute to early identification, classification of disease severity, and better prognostic evaluation of CSVD.

With “cerebral small vascular disease” as the keyword, we systematically identified and analyzed 557 articles from China national knowledge infrastructure (275 articles) and PubMed (282 articles), and there were approximately 33 significant factors affecting CSVD, which were as follows (Table 2): age, hypertension, diabetes, atherosclerosis, low-density lipoprotein (LDL), high levels of homocysteine (HH), serum uric acid (SUA), cell adhesion molecule 1 (CAM1), endothelin-1/nitric oxide (ET-1/NO), vascular endothelial growth factor (VEGF), cystatin C (CysC), neurofilament light chain (NfL), C-peptide (C-P), alkaline phospholipase, lipoprotein phospholipase A2 (LP-PLA2), tumor necrosis factor (TNF-α), interleukin-6 (IL-6), fibrinogen (FIB), thrombomodulin (TM), tissue factor pathway inhibitor (TFPI), prothrombinfragment1 + 2 (F1 + 2), plasminogen activator inhibitor-1 (PAI-1), D-dimer (D-D), lipoprotein a (Lp[a]), 25-dihydroxyvitamin D, C-reactive protein (CRP), ferritin, urinary microprotein-to-creatinine ratio, CSF albumin-to-serum albumin ratio, matrix metalloproteinase (MMP), peroxisome proliferator-activated receptor (PPAR), paraoxonase (PON), APOEε4, and et al. These factors trigger different pathology of CSVD indirectly and play various roles in the prevention, diagnosis, prognosis, and identification of CSVD, which are illustrated as follow:

**Table 2:** Classification of CSVD-related influencing factors.

**Classification**		**Makers**
Biological markers	Markers of Vascular Endothelial Injury	• CAM1
		• VEGF
		• ET-1/NO
		• HH
	Nerve damage markers	• NfL
	Inflammatory markers	• CRP
		• IL6
		• LP-PLA2
		• TNFα
		• FIB
	Oxidative stress markers	• PPAR
		• PON
	Blood-brain barrier injury markers	• MMP
	Cerebrospinal fluid markers	• CSF albumin-to-serum albumin ratio
Risk factors	Independent risk factors	• CysC
		• TM
		• C-P
	Important risk factors	• SUA
		• LDL
		• TFPI
		• D-D
		• Lp(a)
		• F1+2
	Other risk factors	• age
		• hypertension
		• atherosclerosis
		• diabetes
Indicators	Important indicator	• alkaline phospholipase
		• ferritin
	Predicators	• PAI-1
		• 25-dihydroxyvitamin D
		• urinary microprotein creatinine ratio
Genes	Predisposing genes	• APOEε4

### Biological Markers

#### Markers of Vascular Endothelial Injury

CAM1 is an independent risk factor for CSVD, closely related to the progress of WMLs. Activated adhesion molecules bind to the carrier protein on the surface of macrophages and adhere to the surface of endothelial cells (ECs). Vascular endothelial damage is caused by several inflammatory cascades (25). VEGF plays a role in the occurrence, development, and outcome of CSVD cognitive impairment (26), and its increase is speculated to be due to self-protective mechanisms. As for the vascular tension factor ET-1/NO, adjusting and maintaining its balance can ensure the continuous stability of vasodilation, vascular contraction function, structure of the blood vessel wall, protection of brain tissue, slowing down of the progression of CSVD, and the occurrence of VD. HH mainly affects cognitive function through neurotoxic pathological processes and vascular atherosclerosis (27) and is related to its involvement in the decline of frontal lobe executive function and parietal spatial structure ability (28). To some extent, it can be used as a significant predictor of CSVD-related cognitive decline to some extent. In addition, studies have shown that HH caused by EC dysfunction is a significant predictor of ischemic LA in patients with CSVD (29).

#### Nerve Damage Markers

The NfL is a new type of biomarker for nerve axon damage, which is deemed essential in various neurological diseases. Studies have shown that the level of NfL in the CSF is significantly correlated with WMH and can be used as a potential marker of CSVD load (30). In addition, the level of NfL in the urine was significantly correlated with all MRI markers of CSVD. In particular, WMH can be an alternative because of the relationship between the average diffusion rate of MRI and the determination of CSVD after excluding other lesions. It can effectively reflect MRI indicators and the severity of clinical symptoms to a certain extent.

#### Inflammatory Markers

CRP is speculated to directly participate in endothelial dysfunction by inducing the release of cytokines and the surface expression of adhesion molecules (31). In addition, CRP combines with cholinesterase and identifies exogenous pathogens, thus directly participating in the process of ischemic neuronal necrosis, which eventually leads to damage to the neural network and cognitive impairment. Therefore, CRP in the blood can indicate vasculitis, and the degree of atherosclerosis directly and indirectly indicates the severity of VD. Additionally, the IL-6 carrier protein is related to the severity of WML, which may be a marker of chronic inflammation (32). The increase CRP and IL-6 levels are related with asymptomatic cerebral infarction and WML (33). LP-PLA2 plays an important role in various vascular pathological mechanisms such as atherosclerosis and ECs dysfunction by participating in inflammation and LDL metabolism (34). TNF-α leads to neuronal damage by increasing the permeability of the blood-brain barrier (BBB) and accelerates the occurrence of CSVD cognitive dysfunction (35). FIB is an essential regulator of the inflammatory cascade, and plays a key role in coagulation and inflammation. An increase in the FIB concentration can trigger vascular endothelial damage, change blood flow and microcirculation, increase blood viscosity, and cause neurovascular dysfunction (36). FIB also causes intracerebral hypoperfusion that aggravates CSVD and LA (37), and CSVD is strongly linked with cognitive impairment.

#### Oxidative Stress Markers

PPAR binds to DNA regulatory elements and peroxisome proliferators. The compound then binds to retinol X receptors to form heterodimers and regulates the transcription of multiple genes. There are three subtypes of PPAR: α, β/δ, and γ, high level of PPAR-γ are closely related to WML (38). Circulating PON leads to atherosclerosis by inhibiting the peroxidation of high-density lipoproteins and the accumulation of LDL lipid peroxides in the endothelium. The PON54LL genotype can promote the onset and worsen the progression of WML in the aged (39).

#### BBB Injury Markers

MMP is regarded as a research hotspot in recent years. It can decompose basement membrane proteins and tight junction proteins around cerebral blood vessels. These proteins are involved in the neuroinflammatory response, which causes an increase in inducible MMP-3 and MMP-9 levels in the CSF. They can enhance the activity of MMP, thereby aggravating damage to blood vessels and finally entering the blood. When the BBB is destroyed, MMP enter the CSF, causing white matter demyelination. In addition, common polymorphisms of MMP-1, MMP-2, and MMP-3 are linked to VD, especially subcortical VD caused by CSVD. MMP-2 gene-1306T/C polymorphism is an independent risk factor for WML. The level of MMP-9 also correlated with the volume of the WML.

#### CSF Markers

An increase in the albumin-to-serum albumin ratio in the CSF has a negative effect on WML. The increase in the albumin concentration in the CSF of patients with WML indicates that blood albumin enters the CSF through the BBB (40).

### Risk Factors

#### Independent Risk Factors

As an inflammatory marker, CysC is stable and is not affected by age, sex, race, or nutrition of patients (41). Taking this advantage, CysC becomes an independent and potential risk factor for CSVD (42) to reflect the early onset of CSVD, especially WMHs (43). The CysC works in two ways. On the one hand, in the early stage of small atherosclerosis, vascular smooth muscle cells are stimulated to secrete large amounts of cathepsins, thereby improving the hydrolysis of elastic tissue. As an endogenous cathepsin inhibitor, CysC expression decline eventually leads to the accelerated progression of arteriosclerosis (44). On the other hand, CysC can induce platelet adhesion and promote brain tissue (ventricle and cerebral cortex) ischemia and hypoxia, leading to CSVD, further triggering cognitive dysfunction. TM is a membrane glycoprotein synthesized by vascular ECs that can prevent platelet activation, inhibit white blood cell activity, and hamper NF-kB expression. TM is an important marker for EC damage (45). TM is released into the blood after vascular ECs are damaged, and the degree of damage is positively related to blood TM levels (46). C-P can indirectly reflect the concentration of insulin, and abnormally increased insulin content, together with insulin resistance, is an independent risk factor contributing to the development of CSVD (47). C-P levels cause endothelial function lesions in cerebral small vessels and are one of the main causes of cerebral WMH (48).

#### Important Risk Factors

SUA is an important risk factor for CSVD and WMHs (49). Excessive levels of SUA damage vascular ECs and hinder the expression of nitric oxide synthase, which causes endothelial dysfunction. In addition, blood urate produced, along with a high level of SUA, is deposited on the vascular wall to trigger inflammation and oxidative stress in vascular ECs. At the same time, lipoprotein metabolism dysfunction may occur. Vascular endothelial dysfunction leads to white matter and cerebral small blood vessel damage in the brain, thus causing CSVD (50). Therefore, SUA can be used as a key indicator of CSVD for individuals at different stages, especially those aged. Carrying out targeted treatment and care according to the changes in SUA levels can effectively reduce CSVD. Generally speaking, LDL is a risk factor for ischemic stroke (51). The increase in LDL levels is one of the reasons for ischemic stroke. LDL functions in platelet activation and tissue factor expression (52). The decrease in LDL levels causes a decrease in the cholesterol concentration in vascular ECs, which may lead to arterial wall destruction, including fragile arteries, bleeding, and slow repair after bleeding (53). This shows that LDL is a risk factor for hemorrhagic stroke, including CSVD. Therefore, LDL is a fundamental risk factor for CSVD, and it is of great significance to adjust its level reasonably. TFPI is a representative factor for various tissue factor inhibitors. Additionally, it is a coagulation marker. It inactivates Xa in tissue factor VIa/Xa to prevent thrombosis. TFPI has also been used to identify endothelial damage (54). D-D is a fibrin monomer formed by FIB in plasma under the action of thrombin. It is an important indicator of the fibrinolysis system and is primarily used to evaluate the presence of thrombosis and secondary fibrinolysis in the body (55). Multiple studies have shown that D-D levels are significantly increased in patients with CSVD. Additionally, it has a negative impact on LI, LA, and other pathological changes. Elevated D-D levels can lead to microcirculation disorders in the subcutaneous tissues of the brain and induce cognitive dysfunction. FIB, as a coagulation factor, stimulates the aggregation of red blood cells and platelets and has an inhibitory effect on fibrinolysis. The increase in FIB causes an increase in blood protein, so that the blood viscosity increases and the shearing force of vascular EC changes. Its degradation products can also directly damage the blood vessel walls, leading to CSVD. Lp(a) is composed of protein and lipid molecules that act as carriers of cholesterol and similar substances in the blood. As an independent predictor of vascular accidents and a systemic vasculitis marker, an increase in Lp(a) illustrates the aggravation of atherosclerosis (56). In addition, studies have found that Lp(a) is related to CSVD (57) and induces cognitive impairment (58). F1 + 2 is a peptide released during the conversion of prothrombin into thrombin, which is the final step of the coagulation cascade. Therefore, the coagulation status in the cerebral arteries was suggested to be associated with WMH (59). In a report by Kario et al., a high level of plasma F1 + 2 was shown to be a risk factor for stroke in elderly hypertensives (60), which is also an important factor for CSVD.

#### Other Risk Factors

Age, hypertension, atherosclerosis, and diabetes are also important risk factors for CSVD. Among them, CSVD incidence is highly correlated with age and does not vary with sex, race, or geographic environment (61). Hypertension is the most notable but modifiable risk factor for CSVD. A lack of control or irregular control may result in cognitive deficits (62). Atherosclerosis is the core pathological factor leading to CSVD. For instance, the higher the level of LDL, the more severe the cerebral small blood vessel disease, and in terms of neuroimaging, the more evident are the markers of CSVD such as brain microhemorrhage and white matter high-density shadow (51). Insulin is a recognized risk factor for atherosclerosis. Microvascular endothelium is more susceptible to the metabolic and mitogenic effects of insulin than is the large-vessel endothelium. Studies have shown that high blood glucose levels can increase the rate of microglial apoptosis and neuronal damage (63). Moreover, genetic susceptibility to T2D and higher HbA1c levels are associated with the risk of ischemic stroke, large artery stroke, and small-vessel stroke (64). Therefore, the blood glucose level is considered a risk factor for CSVD.

### Indicators

#### Important Indicator

Phospholipid degradation is an important factor that promotes neuronal death after transient cerebral ischemia. Alkaline phospholipases with both phospholipase and lipase activities can degrade phospholipids, promote neuronal apoptosis, and deteriorate cognitive dysfunction in CSVD (65). Therefore, the use of citicoline to weaken phospholipase activity and inhibit phospholipid degradation has been proven to be effective in terms of neuroprotection (66). Ferritin is a neuroprotective protein that can restore pathogenic changes, impair cell viability, increase apoptosis, and increase caspase-9 cleavage in hippocampal neurons (67). Existing studies have found that serum ferritin is an important indicator of cognitive dysfunction (68).

#### Predicators

In terms of predictors of CSVD, first, the imbalance in thrombus fibrinolysis may be one of the main reasons for the prognosis of ischemic stroke. Dynamic detection of biomarkers in plasma may help predict the early prognosis of acute ischemic stroke (69). PAI-1 can reduce fibrinolytic activity, thus causing susceptibility to thrombi. PAI-1 genotypes 4G4G and 4G5G improve the expression of PAI-1 and jointly participate in the onset of ischemic stroke. PAI-1 is also related to stroke severity throughout the disease course (70). Therefore, it is helpful for clinical diagnosis and evaluation of severity. Clinical studies have found that a deficiency in 1,25 dihydroxyvitamin D3 can increase the risk of hypertension, coronary heart disease, and cardiac insufficiency. Its mechanism of preventing cardiovascular diseases may include regulation of the renin-angiotensin system, inhibition of myocardial hypertrophy and myocardial cell proliferation, anti-inflammatory effects, anti-atherosclerosis effects, and vascular protection (71). The urinary microprotein-to-creatinine ratio is a sensitive indicator of early renal damage. Common risk factors, such as coagulation abnormalities, impaired vascular EC function, inflammatory reactions, and uremia-related factors, may occur in patients with ischemic stroke and CSVD, and it has an undesirable effect (72). Furthermore, latest research shows that Aβ-42 combined with the CSVD score can also be used to predict vascular cognitive dysfunction (VCI) in patients with CSVD (73).

#### Gene Markers

Predisposing genes: APOEε4 alleles are associated with Aβ deposition, which may lead to the onset of WML, especially in the frontal lobe (74).

## Pathological Mechanism

### Chronic Ischemia/Hypoperfusion

**Figure 1:**
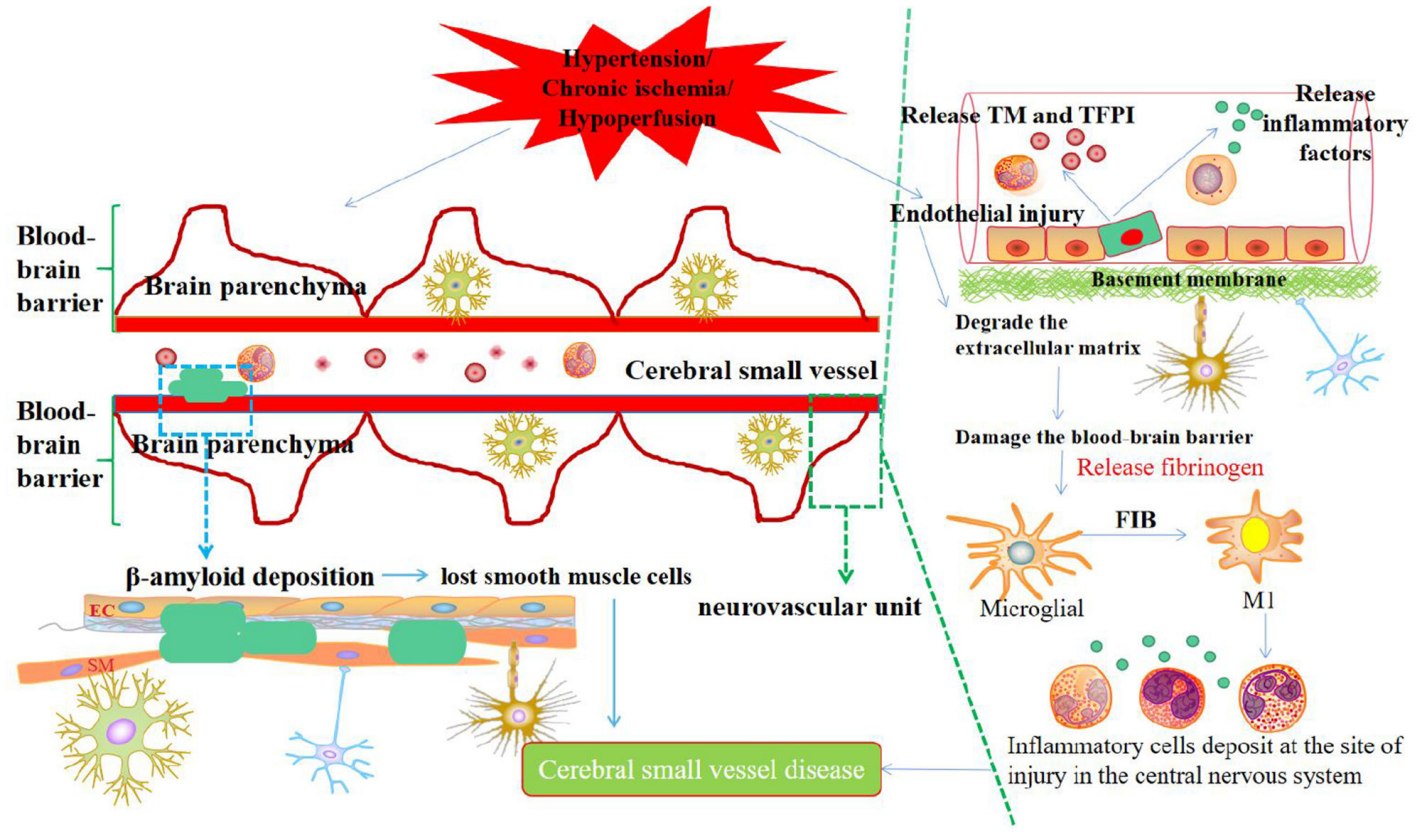
Synthetic diagram of the mechanism of CSVD.

Chronic ischemia or hypoperfusion originates from structural changes in microvessels, deep brain perforating artery injury, decreased cerebral blood perfusion, and selective oligodendrocyte apoptosis. Hypoperfusion and myelin degeneration resulting from thick arterial intima, plaque obstruction of perforating arteries, and vascular lumen stenosis can lead to LA, and acute cerebral ischemia in local brain tissue caused by acute occlusion of small blood vessels might lead to LI. The mechanism may be that cerebral ischemia triggers the release of neuroinflammatory factors, which activates microglia/macrophages to release proteases and free radicals, thereby resulting in permanent damage to the extracellular matrix and neurovascular units, which, in turn, leads to CSVD (Figure 1).

### Atherosclerosis

Atherosclerosis, especially intracranial carotid atherosclerosis, is associated not only with ischemic stroke, vascular cognitive impairment (VCI) and dementia but also with CSVD. The specific causes of atherosclerosis are speculated to be senile diabetes and hypertension (75, 76). The continuous increase in arterial pressure and blood flow load leads to cerebral microvascular injury, which further increases the penetration of the vascular pressure load into the microvascular circulatory system, causes microvascular remodeling reactions, and finally leads to impaired reactivity and microvascular ischemia in blood vessels. In addition, damage to the vessel wall causes expansion of the exterior due to fibrosis to become microaneurysms, proximal lumen stenosis, or obstruction, and, ultimately, impaired autoregulation of small vessels leading to reduced cerebral blood flow and chronic cerebral hypoperfusion (77). Cerebral vascular endothelial dysfunction is a significant indicator of the occurrence and development of atherosclerosis, which occurs several years earlier than macroscopic atherosclerosis and can be used as a predictor of stroke and myocardial infarction (78). However, there is still a lack of consistent evidence to support the relationship between atherosclerosis and CSVD. Hence, their correlation needs to be explored further.

### Endothelial Cell Injury and Blood-Cerebrospinal Fluid Barrier Dysfunction

EC dysfunction and BBB destruction are potential mechanisms of early brain injury in CSVD (79). Asymmetric dimethylarginine (ADMA) is a key factor leading to vascular EC damage. Elevated levels of ADMA in the plasma or CSF can competitively inhibit nitric oxide synthase and reduce the synthesis of the vasoactive substance nitric oxide, resulting in the dysfunction of vascular ECs (80, 81). BBB destruction caused by diffuse vascular EC damage and other factors may be the initiating segment of CSVD (82). Cerebrovascular ECs become more sensitive to the pathogenesis of CSVD. Dysfunction occurs before VCI. Early pathological processes, such as small arterial vascular remodeling, lumen stenosis, thrombosis, and secondary ischemia linked with CSVD, are closely related to each other. The early pathological process can cause cerebral blood flow imbalance and BBB damage, resulting in the exposure of nerve cells to harmful substances, thereby activating brain glial cells and inflammatory reactions, which, in turn, induces brain white matter, nerve axons, and synaptic damage, finally leading to CSVD, VCI, and even dementia. Studies have shown that the coagulation system markers TM and TFPI act on the onset and development of CSVD and have been used to identify endothelial injury (83). Specifically, TM is a membrane glycoprotein produced by vascular ECs. When vascular ECs are damaged, they reflexively stimulate and release TM into the blood, thereby preventing platelet activation and inhibiting white blood cell activity and NF-κB expression (45). The TM level is directly proportional to the severity of vascular EC damage (48). TFPI is a representative tissue factor inhibitor and a coagulation marker. It inactivates Xa in tissue factor VIIa/Xa and hampers thrombosis (84). On this basis, we deduced the process of cognitive impairment in patients with CSVD. There are extensive small blood vessels, capillary EC damage, and microvascular disorders that activate coagulation factors. The activated platelets adhere and aggregate, causing viscosity to increase, which triggers tissue ischemia and hypoxia. Thus, endothelial damage was further aggravated. Consequently, the risk of cognitive impairment increases in patients with ischemic CSVD. At present, the most mature method for evaluating vascular endothelial function is to measure the endothelial-dependent vasomotor response to pharmacological or physiological stimuli; however, there is no gold standard scheme for evaluating endothelial function in cerebral circulation. Vascular endothelial dysfunction is closely related to oxidative stress. The reactive oxygen species produced by oxidative stress can oxidize tetrahydrobiopterin to trihydrobiopterin, leading to endothelial nitric oxide synthase dysfunction. The reduction of endogenous nitric oxide production induces an imbalance of vasoactive substances and vascular endothelial dysfunction, which makes it impossible to rely on the self-regulation ability of cerebral blood flow to ensure adequate blood supply during ischemia and hypoperfusion, finally triggering leukoaraiosis.

BBB is a structural barrier that effectively prevents microorganisms and toxins from reaching the central nervous system. Destruction of the BBB is a potential mechanism of early brain damage in CSVD, aging, dementia, LA, and LI. Specifically, the BBB and neurons together constitute a neurovascular unit, which is composed of blood vessels, tight junctions, astrocyte end-feet, basement membrane, and pericytes. Among them, there are many mitochondria in vascular ECs, which provide energy for the transmission between nerves and blood vessels and affect ion penetration and the metabolic barrier. Tight junctions and adhesion junctions exist between the adjacent vascular ECs. The former is composed of transmembrane protein seal proteins, cytoskeletal proteins, occluding proteins, junction adhesion molecules, and cytoplasmic attachment proteins. It can modify the BBB to damage it and change the type of stroke. This process provides new insights into further targeted treatment. Vascular ECs and pericytes are surrounded by the extracellular matrix. Pericytes are blood vessel wall cells embedded in the basement membrane of capillaries. Pericytes are susceptible to enzymatic degradation, which leads to changes in BBB permeability. In addition, they participate in angiogenesis and eliminate toxic metabolites. Long-term chronic cerebral ischemia can damage the ECs and affect the expression of tight junction proteins, thereby increasing the BBB permeability. Moreover, with the increase in age and the emergence of chronic hypertension, the self-regulation ability of small cerebral blood vessels to changes in blood pressure decreases and arterial stiffness increases, resulting in small cerebral arteries responding to changes by increasing the flow rate and pulsation of blood flow. These changes in the hemodynamics of the BBB can lead to EC damage, and increased blood flow shear stress also changes the permeability of the BBB (85). Therefore, the protein and lipid components in the blood extravasate into the blood vessel wall and the surrounding brain parenchyma, which causes lipid hyaline degeneration of the blood vessel wall and toxic damage to the brain parenchymal nerve cells. CSVD-related imaging and pathological changes were also observed. CSVD severity is related to the severity of WMH and increased BBB permeability of the basal ganglia. The relationship between BBB integrity and CSVD requires further research to elucidate the mechanism of CSVD, identify suitable biological markers, and explore effective treatment methods.

### Immune Inflammatory Response

The inflammatory immune response is involved in various central nervous system disorders. Currently, multiple studies have indicated that the immunoinflammatory response plays a core role in the onset, progression, and recovery of stroke (86) and changes in the neurovascular structure and function of CSVD (87). However, the pathological mechanism underlying the CSVD immune response remains unclear.

At present, the immune inflammatory response factors in CSVD mainly include CRP, IL-6, IL-10, TNF-α, and so on (88, 89). Specifically, these influencing factors affect various pathological mechanisms, such as vascular EC activation, BBB destruction, microglia activation, NF-KB signaling pathway activation, and increase in pro-inflammatory factors. The inflammatory immune response is the driving factor for EC dysfunction and BBB destruction. Hypertension and chronic ischemia/hypoxia are the two major risk factors for CSVD. They can trigger the overexpression and release of microvascular immune cells, inflammatory factors, and related proteases. These substances accumulate during deposition. The immune inflammatory response damages vascular ECs and degrades the extracellular matrix. Therefore, the permeability of the BBB increases to harm the brain parenchymal nerve cells, as mentioned previously. The inflammatory immune response involves complex interactions between EC, microglia, and aggressive leukocytes. After EC dysfunction and BBB destruction, blood factor FIB in the neurovascular unit contributes to the communication between the central nervous system and the immune system, consequently entering the brain parenchyma and is deposited as insoluble fibrin. FIB exposes its hidden epitopes and then transforms into an effective activator of the innate immune response (90), directly activating the innate immune response of the central nervous system. In this process, FIB can induce the activation of microglia, differentiate microglia toward the M1 type, and produce various pro-inflammatory factors and monocyte-macrophage chemokines, and then stimulate peripheral monocytes and macrophages to migrate to the central nervous system and deposit in the injured site, leaving the neurovascular unit in a chronic inflammatory condition, which eventually leads to CSVD. Additionally, exogenous substances such as FIB can recognize and induce the activation of the toll-like receptor (TLR4), which is widely expressed on the surface of microglia. After the activation of the TLR4 signaling pathway, the adaptor protein myeloid differentiation factor is recruited, and then NF-KB is activated or even over-activated. The signal transduction pathway leads to the excessive production of TNF-α, IL-1β, IL-6, and other pro-inflammatory cytokines, which, in turn, aggravates the damage to vascular ECs and the BBB. Persistent neurovascular chronic inflammation and BBB destruction further weaken immune tolerance in patients, allow autoreactive T cells to evade immune tolerance control, and induce an autoimmune inflammatory response in the central nervous system. Subsequent diffuse neuron and oligodendrocyte cell damage eventually causes brain parenchymal pathology and cognitive dysfunction. In the future, the specific mechanism of the immune inflammatory response at the onset of CSVD needs to be carefully investigated to promote the effect of CSVD treatment.

### Amyloid Deposits

CAA is mainly associated with advanced age and is the leading cause of cerebral hemorrhage (91). CAA appears in almost all patients with Alzheimer's disease and is responsible for a large proportion of stroke cases and 64–84.9% of cognitive impairment occurring in the elderly (92, 93). An Autopsy showed that the condition can be detected in 20–40% of the non-dementia and 50–60% of the dementia population (94). However, the exact etiology of these lesions remains unknown. The mechanism may be that the clearance of β-amyloid in the small arteries of the cerebral cortex decreases and its deposition increases, which alters the vascular endothelium, destroys the vascular wall, causes luminal obstruction and ischemia, as well as brain tissue ischemia and hypoxia, finally inducing vascular dysfunction and brain parenchyma damage. In addition, vascular lesions caused by amyloid deposition can trigger symptomatic cerebrovascular dysfunction to a certain extent. The clinical features mainly manifest as vascular rupture, leading to recurrent and multifocal spontaneous intracranial hemorrhage and even dementia after hemorrhage (95). However, the exact cause of such lesions remains unclear. Some researchers believe that amyloid deposits in CAA are related to amyloid produced in smooth muscle cells after early injury (96, 97).

### Genetic Mechanism

Genetic factors rarely influence CSVD. Genetic mutations determine the formation of endothelial deposits, such as glycosphingolipid GB3 in Fabry disease, causing stroke in 24% of patients or even small-vessel infarction in more patients (98). Highly penetrant mutations involving NOTCH3, HTRA1, TREX1, GLA, COL4A2, and FOXC1 (99). Numerous genetic studies have focused on elucidating the hereditary background of familial CSVD. For example, COL4A1 and COL4A2 gene mutations lead to a disorder of type IV collagen α chain synthesis, resulting in changes in the structure of the blood vessel wall, increased fragility, and patients with small brain vascular lesions and ischemic strokes (100). Hereditary CSVD is often clinically presented as a multisystem disorder, and its symptoms, signs, imaging findings, and laboratory examinations are quite distinct from sporadic CSVD. Recent research shows that multiple shared pathways that affect the integrity and functionality of the extracellular matrix seem to play an indispensable role in single-gene inherited CSVD and sporadic CSVD (75). Each pathway participates in different manifestations and subtypes of CSVD. The interaction of these genetic mechanisms with surrounding environmental factors explains why various features of CSVD are observed in sporadic populations. In addition, a dramatically increasing amount of evidence shows that there is a protein aggregation cascade in cerebral autosomal dominant arteriopathy with subcortical infarcts and leukoencephalopathy, indicating that the nodes of the pathway may exceed CSVD and that there may be nodes of pathogenic pathways in neurodegenerative diseases. Further studies on the genetics of CSVD may provide more conclusive evidence of the common pathogenic pathways between monogenic and sporadic disorders.

As mentioned above, chronic ischemia/hypoperfusion, small atherosclerosis, EC, BBB damage, immune inflammatory responses, and genetic factors contribute to the pathogenesis of CSVD. To accurately clarify the pathogenesis of CSVD, cooperation among multidisciplinary teams such as neuropathology, neuroimaging, molecular biology, and genomics, combined with the exploration of suitable animal models will advance the basic research of CSVD. Basic research should be integrated with clinical diagnostic and therapeutic characteristics to facilitate further development of CSVD research.

## Treatment

The treatment of ischemic stroke caused by acute CSVD should refer to the prevention and treatment of acute ischemic stroke, including antihypertensive, thrombolytic, antiplatelet, and lipid-lowering therapies. The treatment of chronic CSVD is mainly focused on VCI, and the treatment drugs are mainly targeted at VD, which mainly include the following: ① cholinesterase inhibitors, such as donepezil, carbalatine, galanthamine; ② non-competitive N-methyl-D-aspartate receptor antagonist memantine. In hereditary CSVD, specific enzyme replacement therapy for febrile disease has been proven to be effective, and the main drug is exogenous gene recombinant α-galactosidase A, including arga-β and arga-α. The potential therapeutic targets of CSVD are as follows: ① immune aging may be related to the occurrence of age-related CSVD; ② reducing the immune inflammatory response may delay the progression of age-related CSVD (101); ③ vitamin B supplementation may delay WMH progression in patients with severe CSVD (102). Controlling the risk factors of cerebrovascular diseases, such as hypertension, diabetes, heart disease, hyperlipidemia, obesity, and smoking, can significantly reduce the mortality and disability rates of patients with CSVD. In addition, the therapeutic effects of Chinese herbal medicine, acupuncture, moxibustion, and guidance on cognitive impairment in CSVD have been confirmed (103–106).

Integrated traditional Chinese and western medicine intervention treatments are the direction of development. In addition, considering the heterogeneity of CSVD, future research should focus on determining individualized treatment plans for patients with VCI.

## Difficulties In Research

### Limitations and Challenges of Diagnosis

First, the lack of visualized CSVD lesion imaging technology and qualitative diagnosis hampers accurate diagnosis (107). The distinct pathophysiological mechanisms of brain parenchymal diseases and vascular injuries hinder precise targeted treatment, even though they have similar manifestations. Second, the diagnostic criteria for CSVD are lacking, and the need for quantitative assessment should be further explored. Furthermore, the severity of the clinical symptoms and neuroimaging findings did not match. Therefore, a combination of clinical symptoms, signs, neuroimaging, neurobiology, molecular genetics, neuroepidemiology, and other research methods should be applied to the quantitative and intelligent diagnosis of CSVD. Hence, accurate etiological identification and classification are urgently needed for CSVD.

### Unclear Pathogenesis of CSVD

As mentioned above, the mechanisms linking CSVD are heterogeneous. The interaction between multiple mechanisms makes the specific pathogenesis unknown (107). Hence, in-depth and systematic research on its pathogenesis needs to be conducted further to contribute to the final pathological picture.

### Limited Interventions for CSVD

Currently, CSVD lacks an effective treatment (108). Traditional Chinese medicine, Western medicine, Chinese and Western medicine, and other treatment methods can only have a relatively limited therapeutic effect from one or a few aspects. At the same time, the treatment of CSVD lacks interventions at different time points, mainly because of the following factors. First, the onset of CSVD is relatively insidious, which causes the best treatment time to be missed. Second, although the symptoms of CSVD are mild, the outcomes of some specific therapies are poorer for cerebrovascular disorders caused by CSVD, and the risk of recurrence is high. Third, CSVD is not only a syndrome but also a series of diseases involving small vessels of the brain. Of these disorders, both ischemic and hemorrhagic diseases are included, which leads to a lack of uniform therapeutic criteria for CSVD owing to the contradictions between them. Fourth, risk factors such as hypertension, diabetes, heart disease, and obesity need to persist for a long time. Low compliance may increase the mortality and disability of CSVD patients. Fifth, awareness of CSVD prevention is insufficient in patients, even among neurologists. Strengthening the education on CSVD is not only helpful for clinical care but also beneficial for early diagnosis.

### Insufficiency of CSVD Animal Model Research

An appropriate CSVD animal model is the key to basic research. At present, many animal models of CSVD have been proposed, but there is still no proper model that can reflect all the pathological changes in the small vessels, BBB permeability, white matter, and other characteristics (109). The lack of animal models has severely hindered the exploration of the pathophysiological mechanism of CSVD at the molecular and cellular levels, which harms the potential preclinical treatment, and the exploration of possible related effects with other diseases. Therefore, building a suitable animal model should be a top priority.

## Prospects

### Innovation of Research Pattern

At present, research on CSVD is still in the preliminary stage, and further exploration of its clinical symptoms, signs, neuroimaging features, and neuropathology has plateaued. Several studies have demonstrated the use of an innovative clinical-neuroimaging-pathology research pattern can clarify the risk factors, pathophysiology, pathogenesis, and preventive treatment of CSVD. This pattern also benefits the detection of the relationship between CSVD, cognitive dysfunction, and neurodegenerative diseases.

### Genetics Research Prospects

At present, genetic research on CSVD has achieved better results, but some domains still have huge potential, which is described in the following two aspects: The first aspect is the importance of research. Clarifying the genetic characteristics of CSVD can improve the accuracy of the diagnosis of rare single-gene hereditary and sporadic CSVD. Moreover, accurate genetic characteristics benefit the elucidation of CSVD pathogenesis, especially sporadic CSVD. The second aspect is the technical feasibility. Whole-genome research is a novel genetic technology (110). Through mutation analysis of the whole human genome, the correlation between genes and explicit characteristics was determined, which is useful for studying CSVD with complex genetic factors. Additionally, with the establishment and development of second-generation genotyping and sequencing platforms, the cost of DNA sequencing exploration has decreased significantly in recent years, which will help to discover rare single-gene mutations that cause CSVD. Moreover, cellular, molecular, and biochemical changes in CSVD animal models with specific genetic mutations can contribute to the understanding of rare single-gene genetic changes in the pathogenesis of common sporadic CSVD. An improved animal model that can express the full human CSVD gene sequence can be helpful for studies on targeted treatments. However, the biological information and mutations in some specific genes need to be further explored to clarify their physiological functions and the role of gene products in the pathological mechanism of CSVD.

### Prevention and Treatment of VCI

CSVD was the most common cause of VCI. Future research on aging needs to emphasize the role of CSVD and VCI from the following aspects: improving practical cognitive tests, identifying VCI-related brain injury biomarkers, carrying out comprehensive studies on the correlation between CSVD and VCI, highlighting the importance of population aging factors in VCI clinical and preclinical research, and clarifying the risk factors for VCI and other aspects.

## Conclusions

In summary, CSVD is a serious chronic clinical disease with significant harm and severe impact. At present, progress has been made in the epidemiology, imaging, pathogenesis, and treatment of CSVD. However, several problems remain. This comprehensive review found that research on CSVD needs to be regarded as a whole. Targeted exploration of CSVD from different viewpoints and a logical combination of novel findings from clinical, imaging, and basic research are required to develop an innovative research approach. In addition, because of the complexity of the pathogenesis of CSVD, as well as the increasing awareness of the importance of hereditary CSVD research, and advancement of its technical feasibility in recent years, it can be determined that hereditary CSVD will be a notable research direction, which may provide in-depth insights into CSVD.

## Author Contributions

BR and YG conceived the idea of this article and supervised the research. BR performed the research, analyzed the literatures, wrote the manuscript, and participated in improving the manuscript. All authors reviewed the manuscript. All authors read and approved the final version of the manuscript.

## Funding

This work was supported by the Chinese Medicine Inheritance and Innovation Talent Project-National Leading Talent Support Program for Traditional Chinese Medicine 2018 (No. 12), the National Key Research and Development Project (grant number 2018YFC1705000), the Youth Science Fund Project (81904131), and Study on pharmacodynamic mechanism of Naoshuantong on ischemic stroke and cerebral small vessel disease (HX-DZM-202206).

## Conflict of Interest

The authors declare that the research was conducted in the absence of any commercial or financial relationships that could be construed as a potential conflict of interest.

## Publisher's Note

All claims expressed in this article are solely those of the authors and do not necessarily represent those of their affiliated organizations, or those of the publisher, the editors and the reviewers. Any product that may be evaluated in this article, or claim that may be made by its manufacturer, is not guaranteed or endorsed by the publisher.

## Glossary

CSVD: Cerebral small vessel disease
VD: Vascular dementia
LI: Lacunar in-farction
WMHs: Whitematter hyperintensities
EPVS: Enlarged perivascular space
CMBs: Cerebral microbleed
CTA: CT angiography
DSA: Digital subtraction angiography
DWI: Diffusion weighted imaging
GRE: Gradient echo imaging
LA: Leukoaraiosis
SWI: Sensitive weighted imaging
UHF: Ultra high field
TA: Texture analysis
TP: Texture parameters
DTI: Diffusion tensor imaging
PSMD: Peak width of skeletonized mean diffusivity
FA: Fractional anisotropy
MD: Mean diffusivity
ASL: Arterial spin labeling
PET: Positron emission computed tomography
CNKI: China national knowledge infrastructure
LDL: Low-density lipoprotein
HH: High levels of homocysteine
SUA: Serum uric acid
CAM1: Cell adhesion molecules 1
ET-1/NO: Endothelin-1/nitric oxide
VEGF: Vascular endothelial growth factor
CysC: Cystatin C
Nflc: Neurofilament light chain
C-P: C-peptide
LP-PLA2: Lipoprotein phospholipase A2
TNF-α: Tumor necrosis factor
IL6: Interleukin 6
FIB: Fibrinogen
TM: Thrombomodulin
TFPI: Tissue factor pathway inhibitor
F1+2: Prothrombin fragment 1+2
PAI-1: Plasminogenactivator inhibitor-1
D-D: D-dimer
Lp[a]: Lipoprotein a
CRP: C-reactive protein
MMP: Matrix metalloproteinase
PPAR: Peroxisome prolifer-ators-activated receptor
PON: Paraoxonase
WML: White matter lesion
ECs: Endothelial cells
BBB: Blood-brain barrier
VCI: Vascular cognitive impairment
TLR4: Toll-like receptor
CAA: Cerebral amyloid angiopathy
CADASIL: Cerebral autosomal dominant arteriopathy with subcortical infarcts and leukoencephalopathy.
